# Exploring Spent Coffee Grounds: Comprehensive Morphological Analysis and Chemical Characterization for Potential Uses

**DOI:** 10.3390/molecules29245866

**Published:** 2024-12-12

**Authors:** Robin Zuluaga, Catalina Gómez Hoyos, Jorge Velásquez-Cock, Lina Vélez-Acosta, Isabela Palacio Valencia, Javier Augusto Rodríguez Torres, Piedad Gañán Rojo

**Affiliations:** 1 Facultad de Ingeniería Agroindustrial, Universidad Pontificia Bolivariana, Circular 1 N 70-01, Medellín 050031, Colombia; robin.zuluaga@upb.edu.co (R.Z.); catalina.gomezh@upb.edu.co (C.G.H.); jorgeandres.velasquez@upb.edu.co (J.V.-C.); lina.velez@upb.edu.co (L.V.-A.); isabela.palaciov@upb.edu.co (I.P.V.); javier.rodriguezt@upb.edu.co (J.A.R.T.); 2 Facultad de Ingeniería Química, Universidad Pontificia Bolivariana, Circular 1 N 70-01, Medellín 050031, Colombia

**Keywords:** spent coffee ground, potential uses, morphological analysis, chemical composition, coffee

## Abstract

The agroindustry generates substantial quantities of byproducts, particularly in coffee production, which yields significant waste, most notably spent coffee grounds (SCGs). This study explores the potential of SCGs as a versatile resource for applications in both food and nonfood sectors. A comprehensive chemical analysis revealed that SCGs consist of 30.2 wt.% cellulose, 25 wt.% hemicellulose, and 12 wt.% lignin. Morphological characterization was performed using field emission scanning electron microscopy (FESEM). Additional analyses included attenuated total reflectance Fourier-transform infrared spectroscopy (ATR-FTIR) and thermogravimetric analysis (TGA). ATR-FTIR identified key polysaccharides and oils, whereas TGA offered insights into the thermal degradation behavior of SCGs, confirming the presence of typical plant cell wall components. X-ray diffraction (XRD) patterns revealed low crystallinity, consistent with SCGs’ amorphous structure. Mineral content was assessed using inductively coupled plasma atomic emission spectrometry (ICP-AES) and atomic absorption spectrophotometry (AAS). The results showed that mineral concentrations in SCGs (per 0.01 kg) were within recommended daily intake limits, confirming their safety for potential human consumption. These findings establish SCGs as a valuable lignocellulosic biomass with applications in composite materials. Additionally, it can serve as an organic soil amendment after fermentation to prevent stress on plants. This approach supports effective waste management and advances resource sustainability practices in the agro-industrial sector.

## 1. Introduction

The annual coffee production for the period 2022–2023 was reported at 168.2 million bags, each weighing approximately 60 kg [1]. From this total production, every ton of green coffee generates approximately 650 kg of spent coffee grounds (SCGs), leading to a global annual SCGs production of approximately 6.5 × 10^6^ tons [2]. Additionally, around 50% of the global coffee production is processed into soluble coffee, with about 2 kg of wet SCGs produced for every kilogram of instant coffee manufactured [3].

Quantifying the average generation rate of SCGs from coffee shops and large coffee chains is complex due to significant variation across countries. For instance, espresso machines require roughly 7–9 g of coffee per cup, and the daily coffee usage in establishments ranges from 0.5 kg to 12 kg. This usage translates to an annual SCGs generation of 0.18 to 4.3 tons per establishment.

Given the vast quantities of SCGs generated annually and the challenges associated with their disposal (primarily due to their high organic content requiring adequate oxygen for proper degradation), various efforts have been undertaken to explore alternative uses. SCGs have been investigated as a raw material in both food and nonfood agro-industries [4], contributing significantly to the circular economy by leveraging their diverse bioactive components for biobased product development [5].

Notable examples of valuable molecules derived from SCGs include extracted oil, which can be converted into biodiesel [6]; phenolic compounds with potent antioxidant properties that may benefit human health [4]; and polysaccharides with strong antimicrobial activity against pathogens such as *Cladosporium cladosporioides* and *Phoma violaceaa* [7]. SCGs have also shown good antioxidant activity when processed using various extraction techniques. Beyond molecule extraction, SCGs have been evaluated as an organic amendment to enrich soil with essential elements such as iron (Fe), manganese (Mn), and zinc (Zn) [8]; as reinforcement for biocomposites using biodegradable polymer matrices like polylactide (PLA) [9]; and as a material for biocomposites suitable for civil engineering applications [10]. In terms of energy utilization, in addition to biodiesel production, SCGs have been explored for developing solid biofuels [4,6].

To maximize these applications and explore new possibilities, thorough characterization of SCGs is essential. The literature provides extensive information on SCGs’ chemical composition [11], infrared spectroscopic properties [2,9], and crystallinity [2,9]. This study aims to supplement this knowledge by offering detailed morphological and chemical data to support future investigations and identify potential food and nonfood applications, as well as to establish a route for molecule extraction.

The SCGs analyzed in this study were sourced from coffee shops using various blends of Colombian coffee varieties. Chemical composition studies focused on determining the content of cellulose, hemicellulose, and lignin through tests for acid detergent fiber (ADF), neutral detergent fiber (NDF), lignin, moisture, and ash. These analyses were complemented by an in-depth evaluation of attenuated total reflectance Fourier-transform infrared (ATR-FTIR) spectra to provide a reliable description of the primary organic components of SCGs.

Additionally, this study evaluated the mineral content of SCGs using inductively coupled plasma atomic emission spectroscopy (ICP-AES) and atomic absorption spectrophotometry (AAS). Morphological structure analysis was performed using field emission scanning electron microscopy (FESEM), while X-ray diffraction (XRD) was employed to determine the crystallinity or amorphous nature of the samples. Crystallinity data, along with thermogravimetric analysis (TGA), can inform potential nonfood applications of SCGs, such as their use as filler or reinforcement in biocomposites or as a source of nanocellulose.

## 2. Results and Discussion

### 2.1. FESEM and Chemical Analysis

**Figure 1:**
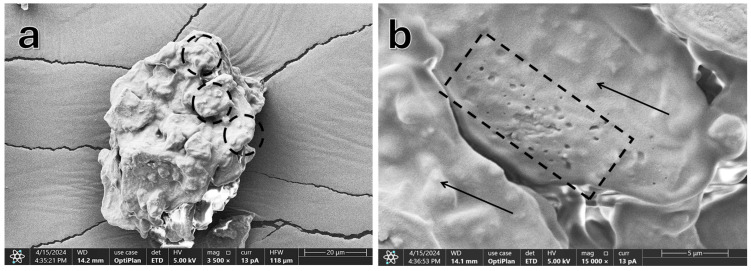
Field emission scanning electron microscopy (FESEM) of SCGs particles. (**a**) Micrograph at 3500× magnification. (**b**) Micrograph at 15,000× magnification.

Figure 1a shows a particle of SCGs exhibiting an irregular shape characteristic of biologically derived materials. Transforming coffee beans into consumer products involves thermal treatments and size-reduction processes that modify their chemical, physical, structural, and sensory properties [12]. Numerous small oil accumulations are visible on the exposed surface and are highlighted with dotted circles in Figure 1a.

The surface reveals segments of the cell wall where polysaccharides in the three-dimensional network have been partially degraded. This degradation occurs during the coffee roasting process at temperatures exceeding 200 °C. At these temperatures, chemical reactions, including the Maillard reaction and pyrolysis, play a significant role [12]. The primary carbohydrates in coffee seed cell walls that transform during roasting include arabinogalactan type II, composed of galactose and arabinose, and galactomannans, which consist of mannans and galactose. Roasting disrupts the cell wall structure, triggering polysaccharide hydrolysis during extraction. This process releases oligosaccharides and monosaccharides, which then degrade into smaller compounds [13].

Figure 1b provides a higher-magnification view, highlighting small oil accumulations (indicated by arrows) and micropores in the cell wall (marked by a dotted rectangle). These micropores reflect structural changes induced by roasting, allowing coffee oil to migrate to the surface of the bean. They vary in size and likely result from the thermal decomposition of organic compounds during coffee roasting, creating voids in the structure. Additionally, the Maillard reaction generates volatile products that escape and contribute to pore formation [12].

Based on the morphological analysis, quantification of the main cell wall components—cellulose, hemicellulose, and lignin—is essential for a comprehensive understanding of the observed structures.

To determine the chemical composition of SCGs, assays for acid detergent fiber (ADF), neutral detergent fiber (NDF), and lignin were performed. The analysis showed that SCGs contain 30.2 wt% cellulose, 25 wt% hemicellulose, and 12 wt% lignin—key components of the plant cell wall’s three-dimensional network. These results classify SCGs as lignocellulosic biomass derived from the nonedible portion of coffee preparation. As such, it does not compete with food resources and holds significant potential for reuse within circular economy strategies. Furthermore, SCGs can be categorized as a tertiary source of cellulose, given their classification as municipal solid waste with a cellulose content comparable to other lignocellulosic materials [14]. Spent coffee grounds (SCGs), a byproduct of coffee consumption, are a potential source of recoverable polysaccharides and phenolic compounds with diverse applications in food and nonfood sectors. In the food sector, SCGs can be classified as dietary fiber under the 2016 definition by the U.S. Food and Drug Administration (FDA), which states that dietary fiber includes “nondigestible soluble and insoluble carbohydrates (with three or more monomeric units) and lignin that are intrinsic and intact in plants; isolated or synthetic nondigestible carbohydrates (with three or more monomeric units) that have physiological effects beneficial to human health” [15].

Recent studies have highlighted the isolation of holocellulose nanofibers from SCGs, rich in mannans, for food applications. These nanofibers exhibit diameters of 2–3 nm and lengths between 0.7 and 1 µm, further underscoring the potential of SCGs in the food sector [16]. These findings show the potential of food based on Pickering emulsion, emulsifiers or stabilizers, or encapsulation of nutrients. To validate the chemical composition of SCGs and support findings from chemical assays, we will analyze this biomass using attenuated total reflectance Fourier-transform infrared spectroscopy (ATR-FTIR).

### 2.2. Attenuated Total Reflectance Fourier-Transform Infrared Spectroscopy (ATR-FTIR)

ATR-FTIR provides structural information about the organic constituents on the surfaces of SCGs by analyzing the reflected infrared signal from the sample. This technique relies on the selective absorption of specific infrared frequencies by molecules, inducing vibrational energy transitions. The resulting spectrum reveals the molecular composition and structural features of the sample, enabling qualitative analysis and structural determination of its compounds.

**Figure 2:**
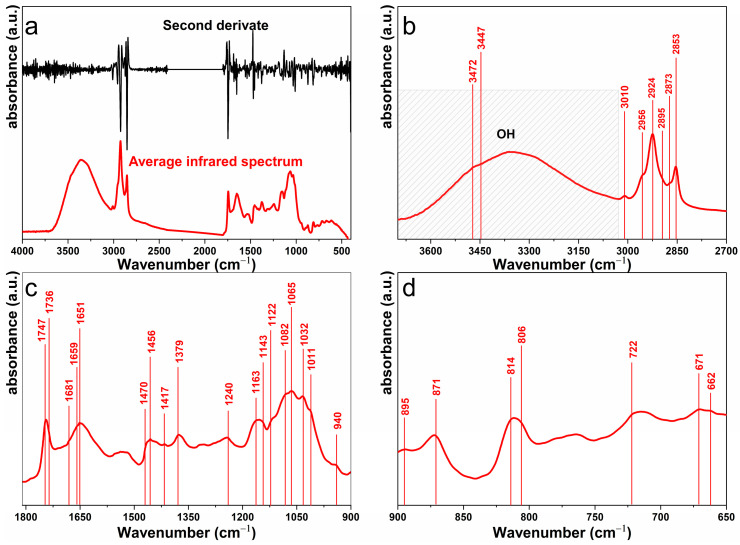
Attenuated total reflectance Fourier-transform infrared spectroscopy (ATR-FTIR) of SCGs is presented as follows: (**a**) The average infrared spectrum (red line) and its second derivative (black line); (**b**) the wavenumber region from 3700 to 2700 cm^−1^; (**c**) the wavenumber region from 1810 to 900 cm^−1^; (**d**) the wavenumber region from 900 to 650 cm^−1^.

Figure 2 displays the ATR-FTIR spectrum of SCGs. Figure 2a shows the complete spectrum in the wavenumber range of 4000–400 cm^−1^, along with its second derivative, which forms the basis for subsequent wavelength-specific analyses.

Zone between 3700 and 2700 cm^−1^

Figure 2b displays the ATR-FTIR spectrum of the SCGs sample and its second derivative in the wavenumber range of 3700–2700 cm^−1^. A broad absorption band between 3700 and 3027 cm^−1^ corresponds to the stretching vibrations of inter- and intramolecular O-H bonds in polysaccharides such as cellulose [17], hemicellulose [18,19], and polyphenolic compounds (e.g., chlorogenic, caffeic, and p-coumaric acids). These polyphenolic compounds are major components of lignin in coffee, as reported by Ballesteros et al. [11] and Grasel et al. (2016) [20]. An overtone of ester carbonyl (C=O) absorption is observed at 3472 cm^−1^, along with the O-H stretching vibration from hydroperoxides at 3444 cm^−1^, previously identified in coffee oils [21].

A minor absorbance at 3010 cm^−1^ is attributed to the symmetric stretching of C-H bonds in cis double bonds (C=C), commonly found in unsaturated fatty acids such as linoleic and oleic acids present in SCGs [21,22,23,24,25]. Shoulder vibrations at 2956 and 2873 cm^−1^ correspond to the asymmetric and symmetric stretching of C-H bonds in the aliphatic CH_3_ group of SCGs oil [21]. Additionally, the 2956 cm^−1^ vibration may also be linked to C-H stretching in caffeine [26].

Absorptions at 2924 cm^−1^ (asymmetric) and 2853 cm^−1^ (symmetric) are associated with the stretching of C-H bonds in the aliphatic CH_2_ group, characteristic of fatty acid structures [21,22]. A smaller peak at 2895 cm^−1^ is attributed to C-H stretching in cellulose [27].

Zone between 1810 and 900 cm^−1^

Figure 2c shows a strong absorbance at 1747 cm^−1^, attributed to ester carbonyl stretching (O-C=O) in triglycerides. This absorbance also corresponds to acetyl and uronic ester groups in hemicelluloses, as well as the ester linkage of the carboxylic group in ferulic acid [19]. Additionally, the absorbance at 1163 cm^−1^ is linked to the stretching vibration of the ester C-O group in coffee oil. These findings align with FESEM observations, which reveal the presence of small oil droplets on the surface [21,22]

The absorbance at 1736 cm^−1^ corresponds to the C=O stretching in molecules such as polyphenol esters, chlorogenic acid, caffeic acid, and p-coumaric acid found in coffee [11,28]. A small shoulder at 1681 cm^−1^ indicates amide I vibrations (C=O and C-N stretching) in proteins [29,30]. Another shoulder at 1659 cm^−1^ is attributed to carbonyl groups in caffeine, which are key in quantitative caffeine analysis [22,26].

The absorbance at 1651 cm^−1^ represents the C=C stretching vibration in cis-olefins (cis RHC=CHR) [31,32] and also corresponds to the C=O stretching vibration of amide I in proteins [33]. The vibration at 1470 cm^−1^ is linked to C-H bending in methyl and methylene groups of phenolic compounds [34,35,36], while the absorbance at 1456 cm^−1^ relates to C-H bending in aliphatic CH_2_ and CH_3_ groups [21,23].

The vibration at 1417 cm^−1^ is associated with CH rocking in cis-disubstituted olefins [21,23], and the absorbance at 1379 cm^−1^ corresponds to symmetric C-H bending in the CH_2_ group of coffee oil [21,23].

At 1240 cm^−1^, the absorbance relates to the C-O-C linkage in cellulose [37,38] and may also involve C-O stretching in the O=C-O group of hemicelluloses [39]. Vibrations at 1163 cm^−1^ are also connected to C-O and C-O-C stretching, with contributions from the OH bending mode of arabinoxylans in hemicelluloses [40]. This absorbance also reflects C-O stretching or O-H bending in the C-OH group of cellulose [41] and the C-OH bending of the phenyl ring in chlorogenic acid [42]. The absorbance at 1143 cm^−1^ corresponds to the C-O-C vibration of arabinose in hemicellulose [43] while a small shoulder at 1122 cm^−1^ is linked to CH bending in cyclohexane structures of polyphenols like chlorogenic acid [42,44].

The absorbance at 1082 cm^−1^ represents the C-O-C linkage in the pyranose ring skeleton of cellulose [45] and the bending of the C-OH bond, influenced by branching in hemicelluloses [46]. The vibration at 1065 cm^−1^ corresponds to C-O and C-C stretching in mannan and galactomannan, key components of hemicelluloses [47]. At 1032 cm^−1^, the absorbance is attributed to C-O stretching, C-C stretching, and C_6_-H_2_-O_6_ vibrations in cellulose [43,47]. The absorbance at 1011 cm^−1^ reflects C-O stretching, C-C stretching, and skeletal vibrations (C2-C3, C2-O2, C1-O1) in pectins [47]. Finally, the absorbance at 940 cm^−1^ corresponds to the arabinose ring vibration in hemicellulose structures [47].

Zone between 900 and 650 cm^−1^

In Figure 2d, the absorbance at 895 cm^−1^ corresponds to the C1-H glycosidic deformation mode, involving contributions from ring vibrations and OH bending in arabinoxylans [40]. It is also associated with C1-H bending in cellulose [43,47,48].

The vibration at 871 cm^−1^ is linked to C1-H bending in mannose-containing polysaccharides [18,47], while the absorbance at 814 cm^−1^ corresponds to C1-H bending in glucomannans within hemicelluloses [43,49].

The absorbance at 806 cm^−1^ is attributed to isomers of chlorogenic acids [42,50] and C1-H bending in arabinans and arabinogalactans within hemicellulose structures [43].

The absorbance at 722 cm^−1^ corresponds to the overlapping of the aliphatic CH_2_ rocking vibration and the out-of-plane vibration of cis-disubstituted olefins [21,23] found in coffee oil. The vibration at 671 cm^−1^ corresponds to deoxy sugars such as fucose [51], and the absorbance at 662 cm^−1^ is linked to the out-of-plane bending of C–OH in cellulose [48].

In conclusion, ATR-FTIR analysis of SCGs identified various components typical of plant cell walls, including cellulose, hemicellulose, lignin, and oil. Significant interactions were observed among polysaccharides such as cellulose, pectins, and the amorphous regions of hemicellulose. The cross-linking of these polysaccharides forms complexes, such as cellulose–xylan, which bind to cellulose microfibrils through hydrogen bonds, contributing to a robust supramolecular structure. Furthermore, the extraction of these polysaccharides and oils has significant potential for use in both the food and nonfood industries, including food based on Pickering emulsion or packaging products.

Understanding the structural components of SCGs is crucial for correlating weight changes with the degradation of specific components. This knowledge facilitates the integration of qualitative analyses, such as ATR-FTIR, with quantitative methods like TGA, highlighting their complementarity. Such integration is essential for optimizing applications in food and nonfood application, such as the development of biobased materials useful for packaging or interleaving films. Thermogravimetric analysis (TGA) of SCGs is performed to complement the morphological and ATR-FTIR analyses.

### 2.3. Thermogravimetric Analysis (TGA)

The thermogravimetric analysis (TGA) of biomass reveals several stages, determined by heating conditions and sample composition. Due to the sample’s heterogeneity and its multiple components, degradation temperatures may overlap during heating. This overlap is reflected in the second derivative (DTG), which displays broad degradation peaks.

**Figure 3:**
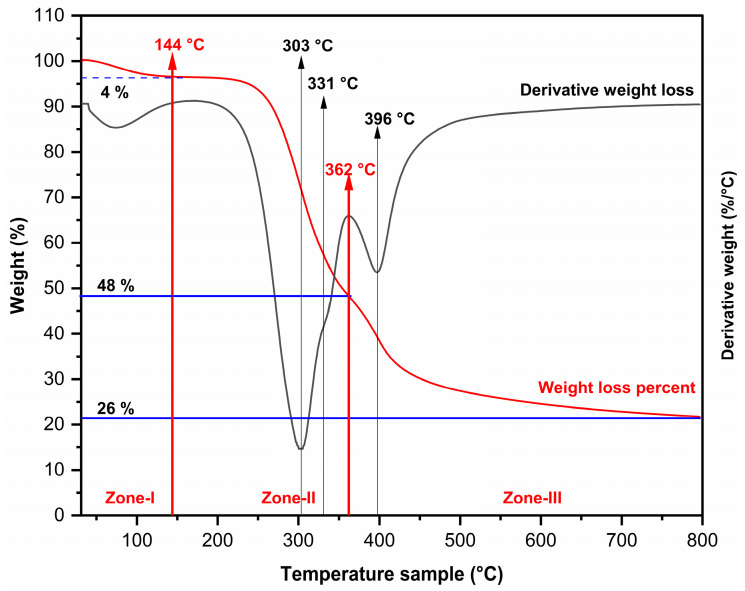
Thermogravimetric analysis of SCGs at a heating rate of 10 °C per minute in a nitrogen atmosphere. Weight loss percent (red line) and derivative weight loss (black line).

Figure 3 shows the TGA curve and corresponding DTG for spent coffee grounds (SCGs). The initial stage involves moisture release, causing a 4 wt% mass loss between 40 °C and 144 °C, with a peak at 74 °C, referred to as Zone I [52,53]. A stable phase follows until 197 °C, after which a significant mass loss occurs, extending to 362 °C and accounting for 48 wt% of the sample. This phase, designated as Zone II, involves the decomposition of cellulose [54], hemicelluloses [55], lignin [53], unsaturated fatty acids, and caffeine [56].

In Zone II, decomposition of biopolymers is accompanied by a secondary water reaction. This reaction results from the dehydration of cellulose, hemicelluloses, and lignin, along with pyrolysis vapors, due to hydroxyl group (-OH) removal [57]. A prominent peak at 303 °C corresponds to the degradation of galactomannans, cellulose, and arabinogalactans in SCGs [11,58,59], which constitute 47 wt% of the sample’s dry weight. Additionally, a shoulder at 331 °C indicates a secondary cellulose degradation stage, caused by rapid depolymerization of glycosidic units, leading to 1,6-anhydro-β-d-glucopyranose (levoglucosan) formation. This reaction predominantly affects the crystalline regions of cellulose, which degrade more slowly than amorphous cellulose [60].

Zone III (362–800 °C), which represents 26 wt% of the sample, exhibits a peak at 396 °C, linked to the decomposition of saturated and unsaturated fatty acids in SCGs oil [58]. In the final stage, between 500 °C and 800 °C, the residual solid decomposes slowly, leading to char consolidation. This stage significantly contributes to the formation of polycyclic aromatic hydrocarbons (PAHs), primarily from chlorogenic acids in coffee grounds, which play a larger role in PAH formation than lignin [61]. The TGA results indicate that this material is suitable for incorporation into polymeric matrices with processing temperatures up to 200 °C, including biodegradable polymers such as PLA [9] and other polymers commonly used in biopackaging development.

The TGA results confirm the presence of structural components typical of plant cell walls, including cellulose, hemicellulose, and lignin. These components were previously quantified using chemical analysis and ATR-FTIR spectroscopy. At the conclusion of the thermal analysis, approximately 22 wt% of residual ash remains, which is primarily composed of minerals. The minerals will undergo further analysis using inductively coupled plasma atomic emission spectroscopy (ICP-AES) and atomic absorption spectroscopy (AAS).

### 2.4. X-Ray Diffraction

**Figure 4:**
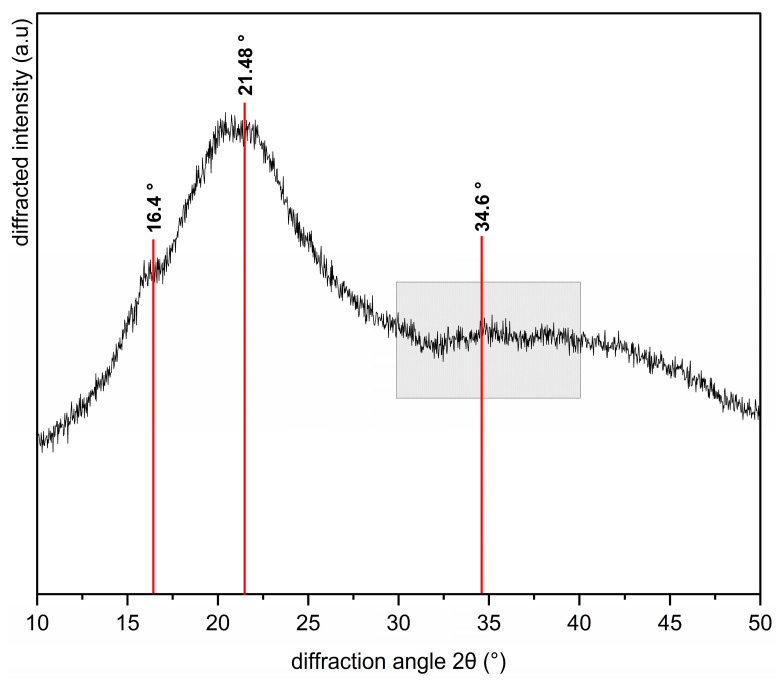
X-ray diffraction (XRD) analysis of SCGs. Amorphous cellulose profiles are associated with the shadow zone in the 30–40° range.

X-ray diffraction (XRD) is a widely used technique for analyzing the crystallographic and chemical structure of cellulosic materials, including natural fibers [62]. Figure 4 shows the XRD pattern of SCGs, which exhibits broad diffraction peaks indicative of low crystallinity, suggesting the presence of small crystalline regions within the sample. The observed pattern reflects contributions from cellulose, hemicellulose, lignin, and oils in the SCGs structure [11]. These findings are consistent with prior chemical characterizations and analyses, including ADF, NDF, total lignin, and ATR-FTIR.

A small shoulder is observed around 16.4°, where arabinan, a hemicellulose polysaccharide [62], converges with the 110 plane of cellulose [63]. A peak at approximately 21.48° reflects intensities converging for hemicellulose polysaccharides, such as arabinogalactans [64] and glucomannans [65], alongside lignin structures [66]. The 30–40° range, highlighted in Figure 4, displays broad intensities indicative of cellulose’s amorphous profile. At approximately 34.6° within this range, the 004 plane of cellulose is identified [67].

The results highlight the predominance of noncellulosic structures in the X-ray diffraction pattern, demonstrating a noncrystalline state with scattering peaks indicative of the sample’s amorphous nature. This characteristic, typical of many lignocellulosic materials, facilitates enzymatic hydrolysis, enhancing the conversion of polysaccharides into fermentable sugars and positioning SCGs as a feedstock for bioethanol production [68,69].

These findings, combined with previous ATR-FTIR and TGA observations, which reveal abundant noncellulosic components and small crystal sizes, indicate that SCGs may exhibit lower mechanical performance as a filler or reinforcing agent for biocomposite materials compared to other natural sources. This assumption stems from the dependency of mechanical strength and stiffness of natural reinforcements on cellulose content and crystal structure [70].

### 2.5. ICP-AES and AAS

The ash analysis of SCGs revealed a content of approximately 6.83 wt% ± 0.95. This result reflects the mineral content, which is quantifiable in greater detail using inductively coupled plasma atomic emission spectrometry (ICP-OES) and atomic absorption spectroscopy (AAS). All higher plants, including coffee, require 16 or more essential elements for their growth [71], primarily sourced from the soil and absorbed through plant roots in inorganic forms. In many cases, these elements do not exist in sufficient concentrations during vegetative growth stages and crop production, requiring fertilizer use. This practice directly influences the production and chemical composition of fruits and other plant tissues.

Table 1 presents the findings of the ICP-AES and AAS investigations, including the calculation of daily intake based on an assumed daily consumption of 1 g. Additionally, it illustrates the percentage contribution relative to the daily value or recommended limit established by organizations such as the European Food Safety Authority (EFSA). None of the analyzed minerals exceed the recommended daily value.

Twenty-two metals were analyzed, among which silver (Ag), boron (B), barium (Ba), cadmium (Cd), chromium (Cr), lithium (Li), nickel (Ni), bismuth (Bi), cobalt (Co), gallium (Ga), indium (In), lead (Pb), and thallium (Tl) fall below the detection limit for the standard matrix available in the laboratory. The results also show the presence of macronutrients such as calcium (Ca), potassium (K), and magnesium (Mg), which are the most concentrated minerals in SCGs, alongside micronutrients such as zinc (Zn), iron (I), copper (Cu), and manganese (Mn). Due to their concentrations, these minerals may be valuable as raw materials for food or nonfood applications rather than for direct use. However, residual caffeine detected in the sample by ATR-FTIR could restrict its use in the food sector, as it may impact human health [69]. For nonfood applications, SCGs could serve as an organic soil amendment at low doses. However, bioactive residues, such as sugars and polyphenols like caffeine, may act as chelating agents, forming complexes with metal ions and reducing macronutrient availability, potentially stressing plants [8]. Using composted SCGs at low concentrations (15% *v*/*v*) is a potential alternative to improve macronutrient bioavailability [72], The degradation of sugars and caffeine before application would reduce the need for organic fertilizers, supporting environmental sustainability.

SCGs can also function as a cellulose source in the form of microfibrils. However, calcium ions (Ca^2+^) can form molecular bonds with pectic substances, creating an “eggs box” structure that decreases pectin solubility and limits microfibril release. Alkaline pretreatments can prevent such structures, facilitating microfibril extraction [73].

Considering the mineral presence in SCGs, it has potential for nonfood applications, particularly as a macronutrient source for soil following fermentation to reduce plant stress. Additionally, it could be used to extract cellulose microfibrils, but minerals that hinder chemical and mechanical processes must be removed.

**Table 1:** Mineral composition spent coffee grounds.

**Inductively Coupled Plasma Atomic Emission Spectroscopy (ICP-AES)**						
**Mineral Element**	**Composition (mg/kg) ^a^**	**Metal Guideline Values (Adults)**	**Parameter**	**Reference**	**EDI (mg/day) ^b^**	**Contribution %**
Aluminum (Al)	27.639 ± 4.025	1 mg/kg bw/week	TWI	[74]	0.028	0.276
Copper (Cu)	23.660 ± 0.405	15 mg/day	UL	[75]	0.024	0.473
Iron (Fe)	35.989 ± 2.949	40 mg/day	AI	[76]	0.036	0.090
Magnesium (Mg)	1106.917 ± 62.165	300 mg/day	AI	[77]	1.107	0.369
Manganese (Mn)	28.357 ± 1.408	3 mg/day	AI	[78]	0.028	0.945
Strontium (Sr)	5.871 ± 0.165	130 µg/kg bw/day	TDI	[79]	0.006	0.065
Zinc (Zn)	32.958 ± 0.807	25 mg/day	UL	[80]	0.036	0.144
**Atomic Absorption Spectroscopy (AAS)**						
Calcium (Ca)	1589.691	2500 mg/day	UL	[81]	1.590	0.064
Potassium (K)	8240.687	3500 mg/day	AI	[82]	8.241	0.235

**Note: bw**, body weight; **UL**, upper intake level; **TWI**, tolerable weekly intake; **TDI**, tolerable daily intake; **AI**, adequate intake. Mean weight of an adult of 70 kg. The Scientific Committee concluded that a default value of 70 kg is a closer approximation to the mean body weight of the EU adult population than the currently applied default value of 60 kg [83]. ^a^ Mean ± standard deviation. ^b^ Supposing a consumption of 1 g/day.

## 3. Materials and Methods

### 3.1. Raw Materials

The coffee shop Laboratorio del Café, located on the campus of Universidad Pontificia Bolivariana in Medellín, Colombia, provided SCGs sourced from at least four different beverage preparation techniques, using a blend of Colombian coffee varieties. The SCGs samples were immediately dried in an oven at 105 °C for 3 h until a constant weight was achieved. The initial moisture content of the samples was measured using a Shimadzu MOC63u moisture analyzer (Kyoto, Japan), yielding a value of 62.04% ± 0.29.

The dried samples were processed using a vertical vibrating sieve shaker, and the material, retained by a 400-mesh sieve (37 µm), was selected for further analysis. These fractions were vacuum-packed in plastic bags using Sammic S.L.’s SV-520 equipment (Sammic, Azkoitia, Gipuzkoa, Spain) and stored at room temperature until testing.

### 3.2. Field Emission Scanning Electron Microscopy (FESEM)

A field emission scanning electron microscope (FESEM) analyzed the microstructure and surface of SCGs. An ion sputter coater applied a gold layer to the samples, which were then examined using a Thermo Fisher Scientific Apreo 2 S FESEM (Thermo Fisher Scientific, Waltham, MA, USA) operated at 5 kV.

### 3.3. Chemical Analysis

The chemical analysis of SCGs powder included the determination of neutral detergent fiber (NDF) treated with amylase, acid detergent fiber (ADF), and lignin (L). These analyses were conducted following the AOAC methods outlined in the animal feed chapter [84]. Cellulose content was calculated as the difference between ADF and lignin (ADF–L), while hemicellulose content was derived by subtracting lignin and cellulose from NDF (NDF–L–cellulose).

Ash content was determined using the ASTM D1102-84 method, a standard procedure for analyzing ash in wood [85].

### 3.4. Attenuated Total Reflection Fourier-Transform Infrared Spectroscopy (ATR-FTIR)

Approximately 1–2 mg of SCGs powder was placed on the crystal for ATR-FTIR analysis. Infrared spectra were acquired using a Nicolet FT-IR iS50 spectrometer (Thermo Fisher Scientific, Waltham, MA, USA) equipped with an attenuated total reflectance (ATR) module. The ATR module featured a type IIA diamond crystal with a 2.8 mm diameter, a 2.0 mm sample area, and a refractive index of 2.4. The single-bounce crystal had an incidence angle of 45°, and a deuterated triglycine sulfate (DTGS) detector was used. The experiments were conducted with a resolution of 4 cm^−1^ and 256 scans.

To enhance comparison with transmission reference libraries, the advanced ATR correction feature in Thermo Fisher Scientific’s OMNIC 9.5.9 software was applied. Each spectrum was smoothed using a 9-point moving average, underwent automatic baseline correction, and was normalized for graphical and qualitative analysis.

Figure 2a presents the complete infrared spectrum of SCGs in the range of 4000–450 cm^−1^. The spectrum represents the average of three individual scans, from which the second derivative was calculated using the Savitzky–Golay filter with a third-order polynomial and a 13-point window [44].

### 3.5. Thermal Analysis (TGA)

The mass loss of SCGs during heating was analyzed using a Mettler Toledo TGA/SDTA 851E thermogravimetric analyzer (TGA) (Mettler Toledo, Greifensee, Switzerland). Approximately 20 mg of SCGs powder was placed in the sample basket for analysis. The sample was heated from room temperature to 800 °C at a rate of 10 °C/min under an inert nitrogen atmosphere.

Differential thermogravimetric (DTG) curves, expressed as % weight/°C, were derived from the thermogravimetric (TG) data, which are expressed as % weight. These calculations provided detailed insights into the thermal degradation behavior of SCGs.

### 3.6. X-Ray Diffraction (XRD)

A Malvern-PANalytical Empyrean 2012 diffractometer (Malvern, Worcestershire, UK) in Bragg–Brentano (powder) geometry analyzed the X-ray diffraction (XRD) of SCGs. For the test, the sample weight was 300 mg of SCGs powder. The instrument operated at 45 kV and 40 mA and used CuKα radiation with a wavelength of 1.5418 Å. A PIXcel3D detector recorded the diffraction, covering a scan range of 10° to 40° 2θ, with an angular step size of 0.0262606°/min and a scan speed of 55 s per step. The sample was positioned on a reflection–transmission spinner rotating at 4 rpm, configured with an omega/2θ goniometer.

The incident beam utilized a 0.04° Soller slit, a 10 mm mask, a 0.5° divergence slit, and a 1° antiscattering slit. The diffracted beam traveled through a 0.04° Soller slit and a 0.5° antiscattering slit, without using a monochromator or collimator. A sample holder measuring 10 mm in diameter and 1 mm in thickness supported the sample.

Background subtraction applied Yao et al.’s method [86], with a zero-background holder made of single-crystal silicon to minimize noise. The analysis corrected XRD data for polarization effects using the factor (1 + cos^2^(2θ))/2 [87].

### 3.7. Inductively Coupled Plasma Atomic Emission Spectroscopy (ICP-AES) and Atomic Absorption Spectroscopy (AAS)

The mineral content in the ashes was determined using inductively coupled plasma optical emission spectrometry (ICP-OES). The analysis was performed with a Thermo iCap 6500 Duo ICP-OES spectrophotometer (Thermo Fisher Scientific, Waltham, MA, USA), following the EPA-3051-A and EPA-6010D methods [88,89]. Quantification of potassium and calcium was conducted using a Thermo ICE 3500 atomic absorption spectrophotometer (AAS) (Thermo Fisher Scientific, Waltham, MA, USA), in accordance with the EPA-3050-B and SM-3111-D methods [90,91].

### 3.8. Evaluation of Dietary Intake

Dietary intake (Equation (1)) was evaluated using the estimated daily intake (EDI, mg/day), assuming a daily consumption of 1 g of product.

(1) $$EDI\left(\frac{mg}{day}\right)=mean\;consumption\;(\mathrm{kg}/\mathrm{day})\;\times \;\mathrm{metal}\;\mathrm{concentration}\;(\mathrm{mg}/\mathrm{kg})$$

After obtaining the EDI value (mg/day), the contribution percentage (Equation (2)) was calculated relative to the recommended value or limit established by various institutions.

(2) $$Contribution\left(\%\right)=\left[\frac{EDI\left(\frac{mg}{day}\right)}{limit\;value}\right]\times 100$$

## 4. Conclusions

This study investigated the chemical characterization and morphological analysis of spent coffee grounds (SCGs) obtained after brewing coffee beverages. Significant findings include FESEM observations, which revealed the irregular morphology of SCGs particles, along with the presence of oil accumulations and micropores in the cell walls. These structural features reflect the impact of thermal treatments and size reduction processes on the chemical and structural properties of SCGs.

Chemical analysis showed that SCGs contain 30.2 wt% cellulose, 25 wt% hemicellulose, and 12 wt% lignin, classifying them as lignocellulosic biomass. This classification highlights SCGs’ potential for reuse in circular economy strategies without competing with food resources. ATR-FTIR analysis identified components typical of plant cell walls, including polysaccharides and phenolic compounds. Significant interactions among polysaccharides suggest potential applications for SCGs in both food and nonfood industries.

Thermogravimetric analysis (TGA) confirmed multiple stages of thermal degradation, indicating the presence of cellulose, hemicellulose, and lignin. The weight loss was attributed to the degradation of these components and moisture release, emphasizing SCGs’ thermal behavior. These characteristics underscores their potential as a filler or reinforcing agent for biocomposite materials, comparable to other natural sources. However, it is important to consider the development of biobased materials that do not require considerable mechanical performance and where the presence of noncellulosic components may improve physical, biological, or chemical characteristics. X-ray diffraction (XRD) patterns revealed low crystallinity, consistent with SCGs’ amorphous nature. This finding suggests that noncellulosic components dominate SCGs’ structure, corroborating prior chemical analyses. Mineral analysis using ICP-AES and AAS revealed that SCGs’ mineral content does not exceed recommended daily values, confirming their safety for human consumption. Accordingly, further research could explore their potential application in food formulations. However, residual caffeine detected by ATR-FTIR could limit their applicability in this sector.

## Data Availability

Data are contained within the article.

## References

[1] International Coffee Organization (2023). Coffee Report and Outlook.

[2] Huang J., Li B., Xian X., Hu Y., Lin X. (2024). Efficient Bioethanol Production from Spent Coffee Grounds Using Liquid Hot Water Pretreatment without Detoxification. Fermentation.

[3] Zhao N., Liu Z., Yu T., Yan F. (2024). Spent Coffee Grounds: Present and Future of Environmentally Friendly Applications on Industries-A Review. Trends Food Sci. Technol..

[4] Jiménez-Zamora A., Pastoriza S., Rufián-Henares J.A. (2015). Revalorization of Coffee By-Products. Prebiotic, Antimicrobial and Antioxidant Properties. LWT.

[5] Ahmed H., Abolore R.S., Jaiswal S., Jaiswal A.K. (2024). Toward Circular Economy: Potentials of Spent Coffee Grounds in Bioproducts and Chemical Production. Biomass.

[6] McNutt J., He Q. (2019). Spent Coffee Grounds: A Review on Current Utilization. J. Ind. Eng. Chem..

[7] Ballesteros L.F., Cerqueira M.A., Teixeira J.A., Mussatto S.I. (2015). Characterization of Polysaccharides Extracted from Spent Coffee Grounds by Alkali Pretreatment. Carbohydr. Polym..

[8] Cervera-Mata A., Navarro-Alarcón M., Delgado G., Pastoriza S., Montilla-Gómez J., Llopis J., Sánchez-González C., Rufián-Henares J.Á. (2019). Spent Coffee Grounds Improve the Nutritional Value in Elements of Lettuce (*Lactuca sativa* L.) and Are an Ecological Alternative to Inorganic Fertilizers. Food Chem..

[9] Wu C.S. (2015). Renewable Resource-Based Green Composites of Surface-Treated Spent Coffee Grounds and Polylactide: Characterisation and Biodegradability. Polym. Degrad. Stab..

[10] Nguyen T.A., Nguyen Q.T. (2021). Hybrid Biocomposites Based on Used Coffee Grounds and Epoxy Resin: Mechanical Properties and Fire Resistance. Int. J. Chem. Eng..

[11] Ballesteros L.F., Teixeira J.A., Mussatto S.I. (2014). Chemical, Functional, and Structural Properties of Spent Coffee Grounds and Coffee Silverskin. Food Bioproc. Tech..

[12] Schenker S., Handschin S., Frey B., Perren R., Escher F. (2000). Pore Structure of Coffee Beans Affected by Roasting Conditions. J. Food Sci..

[13] Oosterveld A., Voragen A.G.J., Schols H.A. (2003). Effect of Roasting on the Carbohydrate Composition of Coffea Arabica Beans. Carbohydr. Polym..

[14] Keijsers E.R.P., Yilmaz G., Van Dam J.E.G. (2013). The Cellulose Resource Matrix. Carbohydr. Polym..

[15] Food and Drug Administration The Declaration of Certain Isolated or Synthetic Non-Digestible Carbohydrates as Dietary Fiber on Nutrition and Supplement Facts Labels: Guidance for Industry. https://www.fda.gov/regulatory-information/search-fda-guidance-documents/guidance-industry-declaration-certain-isolated-or-synthetic-non-digestible-carbohydrates-dietary.

[16] Kanai N., Yamada K., Sumida C., Tanzawa M., Ito Y., Saito T., Kimura R., Saito-Yamazaki M., Oyama T., Isogai A. (2024). Mannan-Rich Holocellulose Nanofibers Mechanically Isolated from Spent Coffee Grounds: Structure and Properties. Carbohydr. Polym. Technol. Appl..

[17] Alghooneh A., Mohammad Amini A., Behrouzian F., Razavi S.M.A. (2017). Characterisation of Cellulose from Coffee Silverskin. Int. J. Food Prop..

[18] Batista M.J.P.A., Ávila A.F., Franca A.S., Oliveira L.S. (2020). Polysaccharide-Rich Fraction of Spent Coffee Grounds as Promising Biomaterial for Films Fabrication. Carbohydr. Polym..

[19] Sun R. (2002). Fractional and Structural Characterization of Hemicelluloses Isolated by Alkali and Alkaline Peroxide from Barley Straw. Carbohydr. Polym..

[20] Grasel F.D.S., Ferrão M.F., Wolf C.R. (2016). Development of Methodology for Identification the Nature of the Polyphenolic Extracts by FTIR Associated with Multivariate Analysis. Spectrochim. Acta Part A Mol. Biomol. Spectrosc..

[21] Raba D.N., Poiana M.A., Borozan A.B., Stef M., Radu F., Popa M.V. (2015). Investigation on Crude and High-Temperature Heated Coffee Oil by ATR-FTIR Spectroscopy along with Antioxidant and Antimicrobial Properties. PLoS ONE.

[22] Dang C.H., Nguyen T.D. (2019). Physicochemical Characterization of Robusta Spent Coffee Ground Oil for Biodiesel Manufacturing. Waste Biomass Valorization.

[23] Vlachos N., Skopelitis Y., Psaroudaki M., Konstantinidou V., Chatzilazarou A., Tegou E. (2006). Applications of Fourier Transform-Infrared Spectroscopy to Edible Oils. Anal. Chim. Acta.

[24] Mota D.A., Santos J.C.B., Faria D., Lima Á.S., Krause L.C., Soares C.M.F., Ferreira-Dias S. (2020). Synthesis of Dietetic Structured Lipids from Spent Coffee Grounds Crude Oil Catalyzed by Commercial Immobilized Lipases and Immobilized Rhizopus Oryzae Lipase on Biochar and Hybrid Support. Processes.

[25] Lauberts M., Mierina I., Pals M., Latheef M.A.A., Shishkin A. (2023). Spent Coffee Grounds Valorization in Biorefinery Context to Obtain Valuable Products Using Different Extraction Approaches and Solvents. Plants.

[26] Gunasekaran S., Sankari G., Ponnusamy S. (2005). Vibrational Spectral Investigation on Xanthine and Its Derivatives—Theophylline, Caffeine and Theobromine. Spectrochim. Acta Part A Mol. Biomol. Spectrosc..

[27] Lan W., Liu C.F., Sun R.C. (2011). Fractionation of Bagasse into Cellulose, Hemicelluloses, and Lignin with Ionic Liquid Treatment Followed by Alkaline Extraction. J. Agric. Food Chem..

[28] Riccucci G., Ferraris S., Reggio C., Bosso A., Örlygsson G., Ng C.H., Spriano S. (2021). Polyphenols from Grape Pomace: Functionalization of Chitosan-Coated Hydroxyapatite for Modulated Swelling and Release of Polyphenols. Langmuir.

[29] Hasanvand E., Rafe A. (2018). Characterization of Flaxseed Gum/Rice Bran Protein Complex Coacervates. Food Biophys..

[30] Sivam A.S., Sun-Waterhouse D., Perera C.O., Waterhouse G.I.N. (2013). Application of FT-IR and Raman Spectroscopy for the Study of Biopolymers in Breads Fortified with Fibre and Polyphenols. Food Res. Int..

[31] Fu X., Su J., Hou L., Zhu P., Hou Y., Zhang K., Li H., Liu X., Jia C., Xu J. (2021). Physicochemical and Thermal Characteristics of Moringa Oleifera Seed Oil. Adv. Compos. Hybrid. Mater..

[32] Suri K., Singh B., Kaur A., Yadav M.P., Singh N. (2019). Impact of Infrared and Dry Air Roasting on the Oxidative Stability, Fatty Acid Composition, Maillard Reaction Products and Other Chemical Properties of Black Cumin (*Nigella sativa* L.) Seed Oil. Food Chem..

[33] Lin H., Bean S.R., Tilley M., Peiris K.H.S., Brabec D. (2020). Qualitative and Quantitative Analysis of Sorghum Grain Composition Including Protein and Tannins Using ATR-FTIR Spectroscopy. Food Anal. Methods.

[34] Abbas O., Compère G., Larondelle Y., Pompeu D., Rogez H., Baeten V. (2017). Phenolic Compound Explorer: A Mid-Infrared Spectroscopy Database. Vib. Spectrosc..

[35] López Pasquali C.E., Herrera H. (1997). Pyrolysis of Lignin and IR Analysis of Residues. Thermochim. Acta.

[36] Shen D., Liu G., Zhao J., Xue J., Guan S., Xiao R. (2015). Thermo-Chemical Conversion of Lignin to Aromatic Compounds: Effect of Lignin Source and Reaction Temperature. J. Anal. Appl. Pyrolysis..

[37] Cao C., Yang Z., Han L., Jiang X., Ji G. (2015). Study on in Situ Analysis of Cellulose, Hemicelluloses and Lignin Distribution Linked to Tissue Structure of Crop Stalk Internodal Transverse Section Based on FTIR Microspectroscopic Imaging. Cellulose.

[38] Marchessault R.H. (1962). Application of Infrared Spectroscopy to Cellulose and Wood Polysaccharides. Pure Appl. Chem..

[39] Stevanic J.S., Salmén L. (2009). Orientation of the Wood Polymers in the Cell Wall of Spruce Wood Fibres. Holzforschung.

[40] Kacuráková M., Ebringerova A., Hirsch J., Hromadkova Z. (1994). Infrared Study of Arabinoxylans. J. Sci. Food Agric..

[41] Nelson M.L., O’Connor R.T. (1964). Relation of Certain Infrared Bands to Cellulose Crystallinity and Crystal Latticed Type. Part I. Spectra of Lattice Types I, II, III and of Amorphous Cellulose. J. Appl. Polym. Sci..

[42] Liang N., Lu X., Hu Y., Kitts D.D. (2016). Application of Attenuated Total Reflectance-Fourier Transformed Infrared (ATR-FTIR) Spectroscopy to Determine the Chlorogenic Acid Isomer Profile and Antioxidant Capacity of Coffee Beans. J. Agric. Food Chem..

[43] Kacuráková M., Capek P., Sasinkova V., Wellner N., Ebringerová A. (2000). FT-IR Study of Plant Cell Wall Model Compounds: Pectic Polysaccharides and Hemicelluloses. Carbohydr. Polym..

[44] Hashimoto A., Mori H., Kanou M., Yamanaka A., Kameoka T. (2004). Mid-Infrared Spectroscopic Analysis on Brewed Coffee Characteristics. Asian Pacific Confederation of Chemical Engineering Congress Program and Abstracts Asian Pacific Confederation of Chemical Engineers Congress Program and Abstracts.

[45] Mehanny S., Abu-El Magd E.E., Ibrahim M., Farag M., Gil-San-Millan R., Navarro J., El Habbak A.E.H., El-Kashif E. (2021). Extraction and Characterization of Nanocellulose from Three Types of Palm Residues. J. Mater. Res. Technol..

[46] Sun R.C., Tomkinson J., Ma P.L., Liang S.F. (2000). Comparative Study of Hemicelluloses from Rice Straw by Alkali and Hydrogen Peroxide Treatments. Carbohydr. Polym..

[47] Liu X., Renard C.M.G.C., Bureau S., Le Bourvellec C. (2021). Revisiting the Contribution of ATR-FTIR Spectroscopy to Characterize Plant Cell Wall Polysaccharides. Carbohydr. Polym..

[48] Liang C., Marchessault R. (1959). Infrared Spectra of Crystalline Polysaccharides. II. Native Celluloses in the Region from 640 to 1700 cm^−1^. J. Polym. Sci..

[49] Chen Z., Hu T.Q., Jang H.F., Grant E. (2016). Multivariate Analysis of Hemicelluloses in Bleached Kraft Pulp Using Infrared Spectroscopy. Appl. Spectrosc..

[50] Barrios-Rodriguez Y.F., Devia-Rodriguez Y., Gutierrez-Guzmán N. (2022). Detection of Adulterated Coffee by Fourier-Transform Infrared (FTIR spectroscopy Associated with Sensory Analysis. Coffee Sci..

[51] Oliyaei N., Moosavi-Nasab M., Tanideh N. (2022). Preparation of Fucoxanthin Nanoemulsion Stabilized by Natural Emulsifiers: Fucoidan, Sodium Caseinate, and Gum Arabic. Molecules.

[52] Parthasarathy P., Narayanan K.S., Arockiam L. (2013). Study on Kinetic Parameters of Different Biomass Samples Using Thermo-Gravimetric Analysis. Biomass Bioenergy.

[53] Singh R., Singh S., Trimukhe K., Pandare K., Bastawade K., Gokhale D., Varma A. (2005). Lignin–Carbohydrate Complexes from Sugarcane Bagasse: Preparation, Purification, and Characterization. Carbohydr. Polym..

[54] Liu C.F., Xu F., Sun J.X., Ren J.L., Curling S., Sun R.C., Fowler P., Baird M.S. (2006). Physicochemical Characterization of Cellulose from Perennial Ryegrass Leaves (*Lolium perenne*). Carbohydr. Res..

[55] Xu F., Sun J., Geng Z., Liu C., Ren J., Sun R., Fowler P., Baird M. (2007). Comparative Study of Water-Soluble and Alkali-Soluble Hemicelluloses from Perennial Ryegrass Leaves (*Lolium peree*). Carbohydr. Polym..

[56] Jin Ong P., Leow Y., Yun Debbie Soo X., Hui Chua M., Ni X., Suwardi A., Kiang Ivan Tan C., Zheng R., Wei F., Xu J. (2023). Valorization of Spent Coffee Grounds: A Sustainable Resource for Bio-Based Phase Change Materials for Thermal Energy Storage. Waste Manag..

[57] Fermoso J., Mašek O. (2018). Thermochemical Decomposition of Coffee Ground Residues by TG-MS: A Kinetic Study. J. Anal. Appl. Pyrolysis.

[58] Li X., Strezov V., Kan T. (2014). Energy Recovery Potential Analysis of Spent Coffee Grounds Pyrolysis Products. J. Anal. Appl. Pyrolysis.

[59] Simões J., Maricato É., Nunes F.M., Domingues M.R., Coimbra M.A. (2014). Thermal Stability of Spent Coffee Ground Polysaccharides: Galactomannans and Arabinogalactans. Carbohydr. Polym..

[60] Cheng K., Winter W.T., Stipanovic A.J. (2012). A Modulated-TGA Approach to the Kinetics of Lignocellulosic Biomass Pyrolysis/Combustion. Polym. Degrad. Stab..

[61] Sharma R. (2004). Characterization of Chars from Pyrolysis of Lignin. Fuel.

[62] Sanjay M.R., Siengchin S., Parameswaranpillai J., Jawaid M., Pruncu C.I., Khan A. (2019). A Comprehensive Review of Techniques for Natural Fibers as Reinforcement in Composites: Preparation, Processing and Characterization. Carbohydr. Polym..

[63] Sugiyama J., Vuong R., Chanzy H. (1991). Electron Diffraction Study on the Two Crystalline Phases Occurring in Native Cellulose from an Algal Cell Wall. Macromolecules.

[64] Wibowo E.S., Park B.D. (2022). Effect of Hemicellulose Molecular Structure on Wettability and Surface Adhesion to Urea–Formaldehyde Resin Adhesives. Wood Sci. Technol..

[65] Kurt A., Kahyaoglu T. (2017). Purification of Glucomannan from Salep: Part 2. Structural Characterization. Carbohydr. Polym..

[66] Goudarzi A., Lin L.T., Ko F.K. (2014). X-Ray Diffraction Analysis of Kraft Lignins and Lignin-Derived Carbon Nanofibers. J. Nanotechnol. Eng. Med..

[67] Montoya-Escobar N., Ospina-Acero D., Velásquez-Cock J.A., Gómez-Hoyos C., Serpa Guerra A., Gañan Rojo P.F., Vélez Acosta L.M., Escobar J.P., Correa-Hincapié N., Triana-Chávez O. (2022). Use of Fourier Series in X-Ray Diffraction (XRD) Analysis and Fourier-Transform Infrared Spectroscopy (FTIR) for Estimation of Crystallinity in Cellulose from Different Sources. Polymers.

[68] Segers B., Nimmegeers P., Spiller M., Tofani G., Jasiukaityte-Grojzdek E., Dace E., Kikas T., Marchetti J.M., Rajic M., Yildiz G. (2024). Lignocellulosic Biomass Valorisation: A Review of Feedstocks, Processes and Potential Value Chains and Their Implications for the Decision-Making Process. RSC Sustain..

[69] Alvira P., Tomás-Pejó E., Ballesteros M., Negro M.J. (2010). Pretreatment Technologies for an Efficient Bioethanol Production Process Based on Enzymatic Hydrolysis: A Review. Bioresour. Technol..

[70] Bledzki A. (1999). Composites Reinforced with Cellulose Based Fibres. Prog. Polym. Sci..

[71] Pilon-Smits E.A., Quinn C.F., Tapken W., Malagoli M., Schiavon M. (2009). Physiological Functions of Beneficial Elements. Curr. Opin. Plant. Biol..

[72] Cruz R., Morais S., Mendes E., Pereira J.A., Baptista P., Casal S. (2014). Improvement of Vegetables Elemental Quality by Espresso Coffee Residues. Food Chem..

[73] Dufresne A., Cavaillé J.Y., Vignon M.R. (1997). Mechanical Behavior of Sheets Prepared from Sugar Beet Cellulose Microfibrils. J. Appl. Polym. Sci..

[74] Aguilar F., Autrup H., Barlow S., Castle L., Crebelli R., Dekant W., Engel K.-H., Gontard N., Gott D., Grilli S. (2008). Safety of Aluminium from Dietary Intake—Scientific Opinion of the Panel on Food Additives, Flavourings, Processing Aids and Food Contact Materials (AFC). EFSA J..

[75] Bresson J.L., Burlingame B., Dean T., Fairweather-Tait S., Heinonen M., Hirsch-Ernst K.I., Mangelsdorf I., McArdle H., Naska A., Neuhäuser-Berthold M. (2015). Scientific Opinion on Dietary Reference Values for Copper. EFSA J..

[76] Turck D., Bohn T., Castenmiller J., de Henauw S., Hirsch-Ernst K.I., Knutsen H.K., Maciuk A., Mangelsdorf I., McArdle H.J., Pentieva K. (2024). Scientific Opinion on the Tolerable Upper Intake Level for Iron. EFSA J..

[77] Agostoni C., Canani R.B., Fairweather-Tait S., Heinonen M., Korhonen H., La Vieille S., Marchelli R., Martin A., Naska A., Neuhaeuser-Berthold M. (2015). Scientific Opinion on Dietary Reference Values for Magnesium. EFSA J..

[78] Agostoni C., Berni Canani R., Fairweather Tait S., Heinonen M., Korhonen H., La Vieille S., Marchelli R., Martin A., Naska A., Neuhäuser Berthold M. (2013). Scientific Opinion on Dietary Reference Values for Manganese. EFSA J..

[79] Pearson A.J., Ashmore E. (2020). Risk Assessment of Antimony, Barium, Beryllium, Boron, Bromine, Lithium, Nickel, Strontium, Thallium and Uranium Concentrations in the New Zealand Diet. Food Addit. Contam. Part A Chem. Anal. Control Expo. Risk Assess..

[80] European Commission (2000). Opinion of the Scientific Committee on Food on the Tolerable Upper Intake Level of Vitamin B 12.

[81] EFSA Panel on Dietetic Products, Nutrition and Allergies (NDA) (2012). Scientific Opinion on the Tolerable Upper Intake Level of Calcium. EFSA J..

[82] Turck D., Bresson J.L., Burlingame B., Dean T., Fairweather-Tait S., Heinonen M., Hirsch-Ernst K.I., Mangelsdorf I., McArdle H., Neuhäuser-Berthold M. (2016). Dietary Reference Values for Potassium. EFSA J..

[83] Antunović B., Barlow S., Chesson A., Flynn A., Hardy A., Jeger M., Knaap A., Kuiper H., Lovell D., Nørrung B. (2012). Guidance on Selected Default Values to Be Used by the EFSA Scientific Committee, Scientific Panels and Units in the Absence of Actual Measured Data. EFSA J..

[84] Latimer G.W., AOAC International (2023). Official Methods of Analysis of AOAC INTERNATIONAL.

[85] (2021). Standard Test Method for Ash in Wood.

[86] Yao W., Weng Y., Catchmark J.M. (2020). Improved Cellulose X-Ray Diffraction Analysis Using Fourier Series Modeling. Cellulose.

[87] Buerger M.J., Klein G.E. (1945). Correction of X-Ray Diffraction Intensities for Lorentz and Polarization Factors. J. Appl. Phys..

[88] Environmental Protection Agency-EPA (2007). U.S. EPA Method. 3051A: Microwave Assisted Acid. Digestion of Sediments, Sludges, and Oils.

[89] Environmental Protection Agency-EPA (2018). U.S. EPA. 2014. “Method 6010D (SW-846): Inductively Coupled Plasma-Atomic Emission Spectrometry,” Revision 4.

[90] Greenberg A., Clesceri L., Eaton A., Franson M.A. (2017). 3111 Metals by Flame Atomic Absorption Spectrometry. Standard Methods for the Examination of Water and Wastewater.

[91] Environmental Protection Agency-EPA (1996). Method 3050B Acid Digestion of Sediments, Sludges, and Soils 1.0 Scope and Application.

